# Suicide Risk Help-Seeking Among Middle- to Old-Age Adults: A Systematic Review

**DOI:** 10.1093/geroni/igac079

**Published:** 2023-01-25

**Authors:** Xiaochuan Wang, Susanny Beltran, Rachael Burns, Marie Hamel, Sydney Gray, Kim Gryglewicz

**Affiliations:** School of Social Work, University of Central Florida, Orlando, Florida, USA; School of Social Work, University of Central Florida, Orlando, Florida, USA; School of Social Work, University of Central Florida, Orlando, Florida, USA; Burnett School of Biomedical Sciences, University of Central Florida, Orlando, Florida, USA; School of Social Work, University of Central Florida, Orlando, Florida, USA; School of Social Work, University of Central Florida, Orlando, Florida, USA

**Keywords:** Suicide, Help-seeking, Service use

## Abstract

**Background and Objectives:**

Suicide has become a major public health concern worldwide and in the United States. Rates of suicide increase during the life course and are highest among middle- to old-age adults. Help-seeking represents a crucial coping mechanism that can mitigate suicide risk. Yet, less is known about suicide risk help-seeking, especially among these age groups. To address this knowledge gap, a systematic review of existing literature was performed to obtain a refined understanding of help-seeking for suicide risk among middle- to old-age adults.

**Research Design and Methods:**

Using Preferred Reporting Items for Systematic Reviews and Meta-Analyses (PRISMA) guidelines, electronic databases and key journals with suicide and/or gerontology focuses were searched to identify peer-reviewed publications in English between 2010 and 2020. A total of 4 732 unduplicated publications were screened for relevance based on titles and abstracts, of which 52 were reviewed in full text.

**Results:**

Twenty-four articles met inclusion criteria and were included in the qualitative synthesis. These articles discussed a range of topics, including the prevalence of service utilization, service use prior to a suicide death, and correlates of help-seeking. In general, the prevalence of service utilization was low and varied by suicidal history (eg, higher prevalence among individuals with a history of suicide attempts than those with suicide ideation only). Key facilitators (eg, current or history of suicidal thoughts, plans, or attempts) and barriers (eg, stigma) for service use and help-seeking were also identified.

**Discussion and Implications:**

Findings highlight the need for future studies and tailored services to improve age-appropriate and culturally responsive suicide prevention and intervention strategies for middle- to old-age adults.

**Translational Significance:** Suicide rates are the highest among middle-aged (ages 45–64) and older (age 75+) adults. This systematic review explores help-seeking for suicide risk, an important yet understudied topic, among middle- to old-age adults. Results indicated a generally low prevalence of service utilization, which varied by suicidal history. The review also identified key service use and help-seeking facilitators (eg, higher suicide literacy, previous or current suicidality) and barriers (eg, stigma). This review underscores the pressing need for future research and tailored services to promote culturally sensitive and age-appropriate suicide prevention and intervention to mitigate the increased suicide risks among this population.

## Background and Objectives

Suicide is a major public health concern. Worldwide, approximately 700 000 people die from suicide each year (1). In the United States, suicide is the 12th leading cause of death (2). Alarmingly, rates of suicide have increased from 10.5 per 100 000 individuals in 1999 to 14.0 per 100 000 individuals in 2020, representing a 33% increase (3,4). Suicide rates are the highest among middle-aged (ages 45–64) females and males aged 75 and older, and rates of suicide increase with age among adults aged 60 and older (3,5,6). Worldwide and in the United States, the population is aging, creating a critical need to address the risk of suicide among middle-aged and older adults.

Aging is associated with an increase in chronic and physical illnesses that often result in functional limitations, disabilities, and pain, including psychological pain, which may be triggered by the loss of autonomy and social isolation (7). Changes in social status and loss of social networks and familial supports (eg, death of a spouse or close relative) are also commonly experienced as individuals age. These life stressors can affect the quality of life and increase the vulnerability of mental health problems and suicide risk (8–10). Indeed, in a recent systematic review, functional disability and specific health conditions such as cancer, chronic obstructive pulmonary disease, neurological disorders, liver disease, male genital disorders, and arthritis/arthrosis were found to be associated with suicidal thoughts and behavior among older adults (11). Moreover, low social integration, lack of social support and belongingness, loneliness, social isolation, and perceived burdensomeness, which may result from changes in social status, bereavement, functional disabilities, and declines in health, have been found to heighten the risk for suicide in aging individuals (9,12–16). Although it is not uncommon for older adults to seek primary care services for medical ailments, suicidal thoughts and intent are rarely communicated or detected by general practitioners in the weeks or months leading to a suicide (17,18). Furthermore, older adults are less likely to initiate and utilize mental health services (19–21). Misconceptions about what constitutes normal aging, ageism, limited knowledge or poor mental health literacy, low perceived need, negative perceptions about treatment, and/or stigmatizing beliefs about mental health may interfere with older adults’ abilities to recognize suicide risk and the need for help (22–25). Cultural factors such as valuing autonomy and self-reliance may also contribute to aging adults’ willingness to engage in help-seeking behaviors (22,25), a key protective factor that may help to reduce suicide risk.

Help-seeking can be viewed as a process that includes problem identification, decisions to seek help, and selection of sources of help (26,27). Help-seeking is an important coping behavior and mechanism by which to find formal and informal support, information, and resources (28). Studies have examined help-seeking for mental health disorders (29,30), such as depression and anxiety; however, limited research has focused on help-seeking behavior for suicide risk, especially among aging populations. The small body of literature that does exist suggests help-seeking for preventing suicide is low, and services are underutilized (22,31–33).

Yet, despite the increasing number of deaths by suicide and high rates of suicide ideation and attempts among middle-aged and older adults (34), little is known about the aging population’s patterns of help-seeking (eg, prevalence, types, and pathways), or the impact of suicide ideation and/or suicidal behavior to service contact and/or use (35,36). Thus, given that help-seeking behavior can mitigate suicide risk and may vary by age among adults (27), it is important to obtain a refined understanding of the various factors that hinder or promote help-seeking behaviors. To address this knowledge gap, the aim of this systematic literature review was to (a) summarize the current knowledge regarding help-seeking for suicide risk among middle- to old-age adults and (b) highlight ways to improve suicide prevention strategies and further research.

## Research Design and Methods

### Data Sources

Using the Preferred Reporting Items for Systematic Reviews and Meta-Analyses guidelines (PRISMA) (37), authors searched databases for peer-reviewed, scientific journal articles exploring and identifying service utilization and help-seeking behaviors for suicide risk among middle- to old-aged adults. The search was an iterative process. The initial search of databases was conducted in January 2020 including ProQuest, EBSCOhost, PsycINFO, JSTOR, Abstracts in Social Gerontology, Sociological Abstracts, and Social Science Abstracts. After consulting subject-matter experts, the search was updated in October 2020 to include additional databases such as PubMed (Medline) and Social Work Abstracts. Searches were limited to publications in English and in scholarly, peer-reviewed journals. The search excluded dissertations, reviews, and other gray literature. Furthermore, key journals with suicide and/or gerontology focuses, such as *Journal of Crisis Intervention and Suicide Prevention*, *Suicide and Life-Threatening Behavior, The Gerontologist*, and *The Journals of Gerontology Series B* were hand-searched to contribute to the overall findings of this systematic review. The iterative nature of the search process helped confirm that the relevant literature had been identified in its entirety, adding to the rigor of the review.

### Search Strategy

Database and journal-specific keywords, such as suicide terms (eg, suicide*, suicidal thoughts), service use and help-seeking terms (eg, help-seeking, service use), and age terms (eg, older adults, mid* adulthood) were used for each database and journal search. The asterisk (*) was used at the end of search terms to retrieve iterations of the root term. A filter was applied to restrict articles published between 2010 and 2020. A total of 4 254 citations were retrieved during the initial search, and another 489 records were identified through the updated search and hand-search of key journals. Titles and abstracts were independently reviewed by 4 authors, and a total of 52 articles appeared from titles and abstracts for full-text eligibility assessment.

### Study Selection

**Figure 1.**
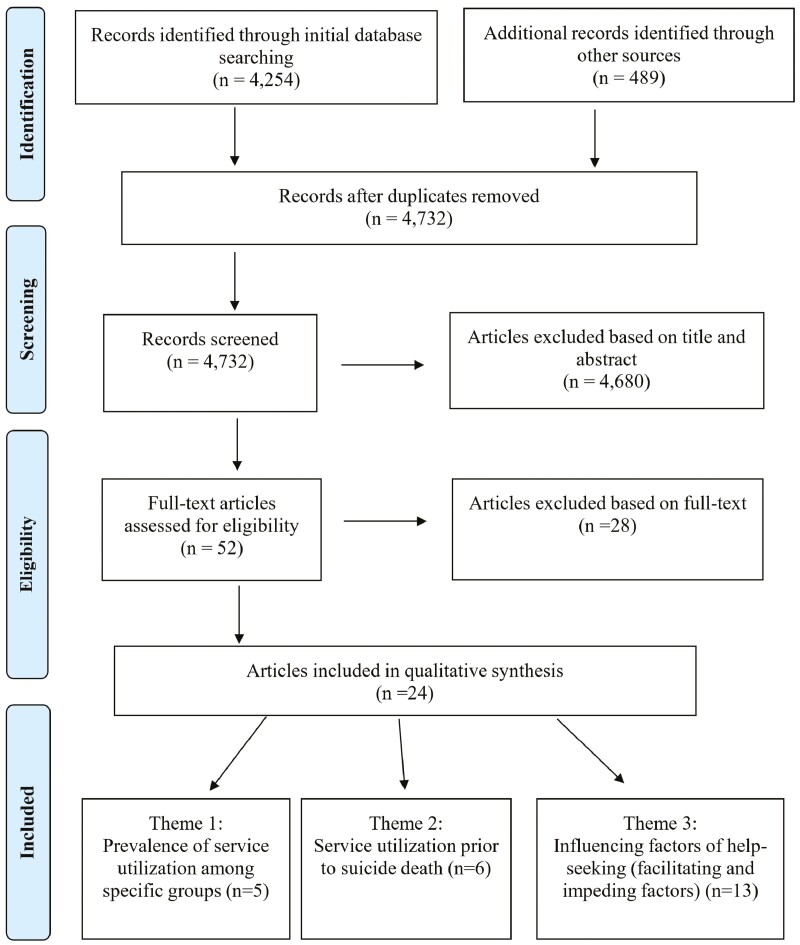
PRISMA flow diagram. PRISMA = Preferred Reporting Items for Systematic Reviews and Meta-Analyses.

Utilizing Cochrane Collaboration’s Data Extraction Template for Included Studies (38) as a guide, an online template was created to document the study selection process. Articles (*N* = 52) were then reviewed independently by 4 authors for inclusion based on the following criteria: (a) publication must have a focus on help-seeking (including attitudes, intentions, or behaviors) related to mitigating suicide risk (eg, defined as having suicidal ideation/thoughts or engaging in suicide-related behavior [suicide attempt, death by suicide]), and (b) publication must specify middle (45–65 years old) and/or old age (65+) adults or identify age as an important factor/finding in the study. When there was disagreement in eligibility rating, authors reread articles and noted additional rationale for inclusion or exclusion. This process continued until there was a consensus among all authors. As a result, a total of *N* = 24 articles were selected to be included in the review. A study selection flowchart of search results is presented in Figure 1. Two authors independently appraised the selected articles and grouped them into preliminary themes based on patterns observed relating to the primary focus of the studies. These preliminary themes were reviewed and discussed with all authors until a consensus was reached as to the labeling of finalized themes. The labeling of the themes captured the essence of the main findings from select articles that met the inclusion criterion. Articles were resorted. The 24 articles were ultimately grouped into 3 themes that describe the main areas of focus across this body of literature.

## Results

### Overview of Publications

The articles (*N* = 24) included in this review utilized a range of study designs and methodologies. The majority (*n* = 22) of studies reported quantitative analyses of primary or secondary data, some of which included nationally representative data sets. One additional article reported the results of an exploratory qualitative study. The remaining article reported both quantitative and qualitative data of a mixed-method study. Among these studies, 9 focused on help-seeking and service use among individuals with suicidal ideation (current/past history) and/or who attempted suicide in the past, 7 focused on service use engagement among suicide decedents, and the remaining (*n* = 8) examined help-seeking behaviors and service utilization with community samples (ie, adults with or without suicidal ideation, prior suicide attempt, or who died by suicide). Studies were conducted in the United States (*n* = 10) and in other countries (*n* = 14). Table 1 provides additional details about the sample characteristics, study design, study aims, and key findings.

**Table 1.** Characteristics of Included Studies (*N* = 24)

Study	Sample	Study Design/Method	Study Aim	Key Findings
Calear et al. (2014)	*N* = 1 274 community sample of Australian adults aged 18 years and older.	Quantitative. Data were collected using online survey.	To investigate the relationships between suicide literacy, stigma, and help-seeking attitudes and intentions.	More positive attitudes and greater intentions for help-seeking were found related to low suicide stigma, high suicide literacy, lower level of depressive symptoms, female gender, and greater age.
Kageyama (2012)	*N* = 4 487 community sample of Japan residents aged 40–74.	Quantitative. Data were gathered using a self-administered questionnaire.	To examine the relationship between views on suicide, demographic factors, and feeling of shame in help-seeking.	Being older (ie, aged 70–74), living in a high suicide standardized mortality ratio or rural areas, holding a pessimistic life view, and viewing suicide as a matter of self-choice, were related to an increased likelihood of feeling shame for suicide help-seeking.
Na et al. (2018)	*N* = 1 200 community sample of Koreans aged 13 and older.	Quantitative. Data were collected through in-person surveys.	To examine the role of age on attitudes toward suicide.	Increased age was found associated with a less favorable attitude toward suicide, which may impede suicide help-seeking.
Blais et al. (2015)	*N* = 2 025 community sample of veterans aged 60 and older (*M*_age_ = 71.0 for full sample, *M*_age_ = 67.6 for distressed subsample).	Quantitative. Data were drawn from the 2011 National Health and Resilience in Veterans Study.	To examine correlates (ie, facilitators and barriers) of current mental health care utilization.	Current suicidal ideation was positively associated, whereas negative beliefs about mental health care and age were negatively associated, with the utilization of services among distressed veterans.
Hom et al. (2016)	*N* = 483 current and retired firefighters aged 18–65 with a career history of suicide ideation, plans, or attempts (*M*_age_ = 36.39).	Quantitative. Data were collected through a national web-based survey.	To identify the prevalence, types, and correlates of mental health service use, as well as barriers to help-seeking, among firefighters with a career history of suicide ideation, plans, or attempts.	Participants reported an overall service use rate of 77%. Firefighters with a history of suicide attempts were more likely to use services than those with ideation only and those with plans but no attempts. Middle-aged participants (ie, 35–44 years and 45–54 years) were at higher odds of service use. In addition, volunteer firefighters, those with shorter service history, serving in small towns or rural areas, with lower incomes, were less likely to utilize mental health services.
Hom et al. (2018)	*N* = 199 women firefighters aged 19–58 in the United States reporting a career history of suicide ideation, plans, and/or attempts (*M*_age_ = 35.87).	Quantitative. Data were collected through a web-based survey.	To determine rates of professional and nonprofessional service use during firefighting careers, and to identify preferred sources, correlates, and barriers to mental health service use.	About 73% of participants reported the use of professional mental health services, and 44% reported the use of other sources of support during careers. Those who were of greater age, with longer service history, and serving as career firefighters (vs volunteer), were more likely to use professional services.
Shaw and Chiang (2019)	*N* = 63 696 at-risk callers to Taiwan National Suicide Prevention Hotline (NSPH).	Quantitative. Secondary analysis of NSPH caller records between 2009 and 2011.	To examine the demographic characteristics of the NSPH callers and to assess the effectiveness of NSPH service in alleviating callers’ emotional distress and suicide risk.	Male and the older (eg, aged 60+), as well as those living outside northern Taiwan, were found less likely to use the NSPH services. Findings indicated that NSPH services were effective in reducing callers’ emotional distress and suicidality.
Han et al. (2016)	*N* = 2 616 Korean adults aged 19 and older with past-year suicidal ideation (*M*_age_ = 48.75).	Quantitative. Secondary analysis using nationally representative cross-sectional data from Korean National Health and Nutrition Examination Survey 2010–2012.	To examine the relationship between socioeconomic factors and mental health service use among people with suicidal ideation.	Service nonuse was found associated with being older (50–64 years, and 65+), widowed, with high school level education, working as a paid employee, absence of depressive mood, with no suicide attempt, and no reported problems in usual activities.
Dey and Jorm (2016)	*N* = 16 640 community sample of Swiss youth and adults aged 15 and older.	Quantitative. Secondary analysis using data from the 2012 Swiss Health Survey.	To examine the presence of help-negation using a Swiss sample.	Few participants with the highest level of suicidality engaged in treatment. Compared to nonsuicidal people, those who experienced suicidality for several days were found to be more likely to engage in treatment. The role of age on treatment engagement was identified but varied by gender.
Ko et al. (2019)	*N* = 1 414 community sample of working-age adults aged 26–64 with reported past year suicidal ideation.	Quantitative. Secondary analysis of 2011 and 2012 National Survey on Drug Use and Health data.	To identify the factors associated with help-seeking among working-age adults reporting suicide ideation.	Factors related to not seeking help included: male gender, being non-White, working full-time, without health insurance, and with lower mental health needs.
Corna et al. (2010)	*N* = 12 792 community sample of adults in Canada aged 55 years and older (*M*_age_ = 67).	Quantitative. Secondary analysis of The Canadian Community Health Survey 1.2: Mental Health and Well-being, nationally representative, cross-sectional data	To estimate the lifetime and 12-month prevalence of suicide ideation among community-dwelling older adults, and examine the association between suicidal thoughts and mental health problems (ie, major psychiatric disorder) and service use.	Less than half of participants with reported past-year suicide ideation accessed any mental health care. Being older, male, had higher social support were associated with a decreased likelihood of mental health service use. Whereas the presence of psychiatric disorders and suicidal thoughts were associated with increased odds of service use.
Vasiliadis et al. (2013)	*N* = 2 004 community sample of French-speaking older adult (aged 65 and older) population living in Quebec.	Quantitative. Secondary analysis using data from Enquête sur la Santé des Aînés, a cross-sectional survey conducted in 2005–2008	To investigate gender-specific determinants of antidepressant and mental health service use for suicidal ideation.	Prevalence rates of mental health service use and antidepressant use were lower among male participants with suicidal ideation, relative to female counterparts. Among these suicidal ideation-reporting participants, the presence of depression was found related to both mental health service use and antidepressant use. Additionally, younger age was found associated with a greater likelihood of antidepressant use.
Mackenzie et al. (2010)	*N* = 3 017 community sample of adults aged 55 and older.	Quantitative. Secondary analysis using nationally representative data from Collaborative Psychiatric Epidemiologic Surveys	To investigate correlates of older adults’ perceived need for mental health services (ie, sought professional mental health services, and with perceived need but didnot seek/receive help).	The lowest level of perceived need was found among participants aged 65 and older and highest among those aged 25–44. Among those with perceived needs for help, greater age (aged 75+ vs 55–64 years) was found to be positively related to help-seeking.
Sheehan et al. (2018)	*N* = 17 338 community sample of adults who reported suicidal ideation and/or behavior during past 12 months. Average age was 26–34 years.	Quantitative. Secondary analysis using nationally representative data from National Survey on Drug Use and Health, 2009–2016.	To investigate racial/ethnic differences in mental health service use among suicidal adults at varied levels of suicidal severity and clinical care intensity.	Past year mental health service use rates were approximately 47% among individuals with severe suicidal ideation (but no attempts) and 55% among suicide attempters, respectively. Racial/ethnic differences were more pronounced in outpatient care, in relative to inpatient care, and varied by suicidal history (ie, suicidal ideators with no attempt vs suicide attempters).
Kisely et al. (2011)	*N* = 108 death records from Nova Scotia Medical Examiner Service (NSMES; *M*_age_ = 44.73).	Quantitative. Data were extracted from the NSMES for suicide cases in 2006 and linked to the provincial administrative databases.	To assess and compare sociodemographic characteristics of people who sought professional help or disclosed intent before suicide, with people who did not.	Seventy-six participants made contact with health professionals, and 46 disclosed suicidal intent, in the year prior to suicide. Adults aged 40–49 had the highest number of suicide cases in the sample, but were least likely to disclose suicidal intent.
Byers et al. (2016)	*N* = 1 139 community sample of adults aged 18 and older with prior suicidal behavior and current mood or anxiety disorders (*M*_age_ = 38.6).	Quantitative. Data were drawn from the nationally representative Collaborative Psychiatric Epidemiology Surveys (2001–2003).	To estimate the prevalence of current (ie, during past 12 months) mental health service utilization by adults with prior suicidal behavior and current mood or anxiety disorders and to examine differences in use by racial-ethnic, age, and gender.	In general, the service use rate was low (<50%). Service use rates were highest among middle-aged adults, compared with the younger and older. African Americans had the lowest use in relative to other racial/ethnic groups. Yet, service use by African Americans increased across the life course, while other groups showed a decrease in service use with older age.
Stanley et al. (2015)	*N* = 2 126 for past-year suicidal ideation, *N* = 690 for suicide plans, and *N* = 345 for suicide attempts, from a community sample of adults aged 18 and older.	Quantitative. Data were derived from the 2013 National Survey on Drug Use and Health.	To identify factors associated with mental health service use among adults with past-year suicidality (ie, ideation, plans, or attempts).	About half of suicidal adults reported past year use of any type of mental health services. Being female, non-Hispanic Whites, with poorer general medical health, and greater severity in mental health clinical profile, were associated with increased likelihood of past-year service use.
Forma et al. (2017)	*N* = 1 118 suicide decedents (*M*_age_ = 77.5), and *N* = 304 955 natural death decedents (*M*_age_ = 83.1), who died in Finland at the age of 70 or older during 1998–2008. *N* = 222 967 individuals who lived longer (*M*_age_ = 81.9).	Quantitative. Secondary data analysis of nationwide register data in Finland.	To analyze the use of health and social services and purchase of medicine during the last 2 years of life among suicide decedents, and compare service use and medicine purchase with that of those who died of natural causes and those who lived longer.	Most (80% or above) older suicide decedents used health (hospital care) or social care (residential homes, sheltered housing, and home care) during last 2 years of their lives. Suicide decedents used less hospital and long-term care, but more psychotropic medication purchase, compared to natural death decedents, adjusting for morbidity. Home and long-term care were used more commonly among older suicide decedents (aged 80+) than the younger (aged 70–79).
Leavey et al. (2016)	*N* = 399 cases of suicide recorded by the Northern Ireland Coroner Service in 2007–2009 (*M*_age_ = 39.16).	Quantitative. A retrospective cohort analysis of deaths recorded as suicide.	To investigate predictors of suicide decedents’ service contact (ie, with health and social care services) in the 12 months prior to suicide and of general practitioners’ vigilance to suicidality.	Over 80% of suicide decedents contacted General Practice services in the 12 months prior to suicide, and the majority of these consultations were attended for mental health problems. Age (35–54 years) was identified as one significant predictor of general practice service contact for mental health problems at 1, 3, and 12 months prior to suicide.
Fontanella et al. (2017)	*N* = 1 338 decedents aged 19 to 65 died by suicide in 2008–2013.	Quantitative. Data were extracted from death certificate files and linked to Ohio Medicaid claims files.	To describe clinical profiles and service use of suicide decedents enrolled in Ohio Medicaid.	Eighty-three percent of decedents made one or more general medical and/or mental health visits during their last year prior to suicide. Decedents with mental disorder diagnoses, substance use disorder, and/or chronic health conditions, as well as those who were older (30–49 and 50–65) and female, were more likely to use service in the last month of life. However, older decedents were less likely to make mental health-only visits during the last month.
Liu et al. (2012)	*N* = 4 406 suicide decedents over age 15 in Taiwan that died in 2006.	Quantitative. Data were obtained from Death Certificate Data file and linked to National Health Insurance beneficiary registry file.	To analyze the characteristics of suicide decedents in their use of outpatient visits and health care during the last year of life.	Rates of a visit by suicide decedents during the last year of life were 85% for overall outpatient services and 30.2% for mental disorders service only. Females were more likely to have outpatient care visits for mental disorder purposes. Individuals 65 years and older had the highest overall rate of visits (97.1%), but the lowest rate for mental disorder visits (25.8%), compared to other age groups.
Waitz-Kudla et al. (2019)	*N* = 267 suicide decedents. Aged 13–80 (*M*_age_ = 36.19).	Quantitative. Data were collected from loss survivors through Qualtrics surveys.	To examine the association between lifetime mental health help-seeking and religious and political beliefs among suicide decedents.	Seventy-five percent of decedents have sought help (ie, taking medication and/or seeing a provider for any mental illness). Suicide decedents with conservative political views (vs those with liberal views) were at a decreased likelihood to seek mental health help before death.
Mallon et al. (2019)	*N* = 403 suicide decedents in Northern Ireland died between 2007 and 2009. This study focused on the male cohort (*n* = 325).	Mixed methods. Data were drawn from Coroner’s records and linked to general practice records.	To examine cases of suicide with no recent (ie, last year of life) health care contact, and to identify factors related to not seeking help.	Eighteen percent of male decedents did not seek support from general practitioners during the last year of life. Older decedents (aged 45–64 and 65+) were more likely to have sought help, compared to the younger.
Troya et al. (2019)	*N* = 16 community sample of participants, including 9 older adults aged 60 who self-harm and 7 third-sector support workers in England.	Qualitative. Data were collected through semistructured interviews.	To examine factors (ie, facilitators and barriers) associated with accessing primary care.	Study identified 3 domains of barriers, including internal (eg, stigma), practical (eg, mobility restrictions), and external (eg, attitudes of healthcare professionals). Key facilitators included older adults’ internal factors (eg, health status and previous positive care experiences), external (eg, providers’ empathy), and structural (availability of regular and continuous support).

### Emerging Themes

The selected studies focused on a range of topics relating to help-seeking behavior and service use among middle- to old-age adults with suicidal ideation and/or who engaged in suicide-related behaviors (including fatal self-harm behavior). Based on their foci, studies were categorized into 3 overarching themes: (a) prevalence of service utilization among specific groups, (b) service utilization prior to suicide, and (c) factors associated with help-seeking.

#### Prevalence of service utilization among specific groups

Five articles reported on the prevalence of service utilization related to suicide among specific groups. Three of these studies reported quantitative analysis results using secondary data from nationally representative surveys (35,39,40). The remaining studies (*n* = 2) used primary data collected on first responder behavioral health, including 1 study that focused on career and volunteer firefighters (41) and another on women firefighters (42). Four of the 5 studies were conducted in the United States, and 1 in Canada.

The 3 studies using national data reported relatively low service use rates. In the study that examined suicide ideation and service use among community-dwelling older adults in Canada, Corna et al. (35) found that over 2% of their sample reported past year suicide ideation, yet less than half of those who reported suicidal thoughts used any type of mental health care. Similar prevalence of service use was found in 2 other studies based on U.S. national data of suicidal adults. Utilizing data from Collaborative Psychiatric Epidemiology Surveys, Byers et al. (39) found a low overall rate (47.3%) of current mental health service use (ie, during past 12 months) among adults with a history of nonfatal suicidal behavior and current mood or anxiety disorders. Rates were lowest among African Americans in the sample (32.2%). Sheehan et al.’s (40) research, based on the 2009–2016 National Survey on Drug Use and Health, also documented relatively low rates of mental health service use among suicidal ideators (ie, those who had severe suicidal ideation but with no attempt history) and suicide attempters (47.4% vs 55.0%, respectively). Non-Hispanic White suicide ideators and attempters were more likely to engage in services at both the inpatient and outpatient level. The remaining 2 studies focused on first responder (ie, firefighters) help-seeking behavior with workers who had a history of suicide ideation, plans, and/or attempt (41,42). In both studies, over 70% of participants reported receiving mental health treatment during their careers; however, specific service use rates varied by different suicidal history (eg, service use rates were higher among firefighters with a history of suicide attempt, compared with those with a plan but no attempt and those with ideation only).

Age was found to be associated with the likelihood of service use, with mixed results. In the national study focused on Canadian older adults, Corna et al. (35) found that increased age was associated with a decreased likelihood of seeking mental health care within a 12-month period. Conversely, in Byers et al.’s (39) study, middle-aged adults had the highest use of mental health services (past 12-month; 52.6%) compared to both younger (41.9%) and older adults (43.8%). Interestingly, racial/ethnic differences in service use emerged across the life course. For example, African Americans, despite lowest service use overall compared to other racial/ethnic groups, showed significant increases in the use of services in older age, whereas Whites, Hispanics, Asians, and other races had a decline in service use with greater age. Other studies showed increased patterns of service use among middle-aged adults (aged 35–44 years and 45–54 years) (41), or with greater age (42). When examining the type of treatment and suicide risk severity, suicidal ideators aged 50 or older were at an increased likelihood of receipt of both inpatient and outpatient services compared to younger adults (ages 18–25); however, older suicide attempters were less likely to utilize outpatient treatment than younger-to-middle-aged counterparts (40).

#### Service utilization prior to suicide

Six studies focused on suicide decedents and retrospectively examined their service use prior to suicide. Specifically, 4 articles reported on suicide decedents’ service use up to 1 year prior to suicide (43–46), 1 study reported on service use during the last 2 years of the decedents’ life (47), and another examined loss survivors’ knowledge of whether and when decedents sought help for mental health concerns at some point during lifetime and prior to death (48). Most of the articles (*n* = 5) in this category reported quantitative analysis results (43,44,46–48); 1 study reported mixed-methods findings (45). Studies were conducted in the United States (*n* = 2), Ireland (*n* = 2), Finland (*n* = 1), and Taiwan (*n* = 1). Only 1 study focused on older adults exclusively (47); the remaining (*n* = 5) examined suicide decedents across age groups.

All of the studies reported that the majority of suicide decedents sought help in their last years of life. The prevalence of service contact or utilization ranged from 75% (48) to 87% (44). A range of service contact and/or utilization were examined and reported in these studies. For example, Forma et al. (47) reported on health (ie, hospital inpatient care) and social services (ie, residential homes, sheltered housing, and home care). Leavey et al. (44) focused on general practice services while also examining other services, such as nursing, social work, accident, and emergency services. Fontanella et al. (43) examined general medical, mental health, and substance abuse treatment visits. In the study conducted by Waitz-Kudla et al. (48), loss survivors were asked to specify suicide decedents’ help-seeking behavior which included taking medication and/or seeing health professionals for mental health issues. Mallon et al. (45) examined suicide decedents’ contact with general practice or psychiatric services in the final year of life. The work by Liu et al. (46) reported on outpatient service use, with a breakdown of the primary reason for the visit (ie, mental vs nonmental disorders). Both Fontanella et al. (43) and Waitz-Kudla et al. (48) explored the role of race/ethnicity (non-Hispanic Whites vs non-Whites) on service use and reported no significant differences.

Four studies identified age as a significant factor of service contact or utilization. For instance, age (ie, middle age, particularly 35–54 years) emerged in 1 study as being a significant predictor of general practice consultation for a mental health problem at 1, 3, and 12 months before suicide, compared with individuals aged 35 or younger (44). Suicide decedents, aged 30–49 and 50–65, were also found to be at an increased likelihood of having general medical or mental health visits in the last month of life, compared to younger adults (43). Yet, older decedents were less likely to use mental health only services. Additionally, Mallon et al. (45) found that individuals of older age groups (aged 45–64 and 65+) were more likely to have utilized primary care services during the last year preceding death, yet such difference did not reach statistical significance. Notably, Liu et al. (46) reported that compared to other age groups, suicide decedents aged 65 or older had the highest rate of overall outpatient visits but the lowest rate of outpatient visits for mental health problems or suicide risk concerns during their last year of life.

#### Factors associated with help-seeking

A total of 13 studies focused primarily on reporting factors that influence help-seeking attitudes, intentions, and/or behaviors. Most articles (*n* = 12) represented quantitative studies, including 4 based on primary data. The remaining article reported the results of an exploratory qualitative study. Four of the 13 studies took place in the United States, 2 in Canada, 2 in Korea, 1 in Australia, 1 in Switzerland, 1 in England, 1 in Japan, and 1 in Taiwan. Of the 13 articles, 4 reported on older adults specifically, with varying foci of population, including Canadian community-dwelling older adults, U.S. veterans aged 60 and older, U.S. adults aged 55 or older, and English older adults (25,49–51). One additional article focused on middle-aged and older populations (40–74 of age) in Japan (52).

These studies identified several facilitating factors that increased help-seeking behavior, including female gender, higher suicide literacy, presence of or higher level of mental health issues, and current or previous suicidal ideation, plan, or attempt. Specifically, studies found that females were more likely than males (27,32,51,53,54) to seek help for suicide risk. Those with higher suicide literacy were found to have more positive attitudes and greater intentions to seek help (32). Additionally, the odds of seeking help for suicide risk increased among individuals with mental health problems such as depression or depressive mood (51,55,56), or who had clinically poorer mental health status (54). Mackenzie et al. (25) found that specific disorders (ie, anxiety, mood, or substance disorders) and the number of disorders experienced also predicted service use. Other key facilitating factors included the history or current presence of suicide ideation (49,51), suicide attempt (56,57), and suicidal behaviors, including ideation, plan, or an attempt (25). Suicidal ideation and/or engagement in suicide-related behaviors that occurred for several days within a 2-week period particularly increased the use of mental health services, compared to individuals without such experiences (55).

On the other hand, several studies examined impeding factors to help-seeking and identified stigma and other attitudinal factors as common and significant barriers. Specifically, greater stigma toward suicide decedents was found related to a more negative attitude and lower odds of seeking help (32). In Mackenzie et al.’s study, participants expressed concerns that their engagement in treatment may affect how they are viewed by others (25). Troya et al.’s study identified stigma related to self-harm and mental health as a key barriers that may delay help-seeking and accessing care (50). Viewing suicide as a matter of self-choice and having a desire of handling the problem on one’s own may also hinder help-seeking (25,52). Negative experiences or dissatisfaction with accessing services (eg, viewing treatment received as ineffective or insufficient, misconceptions about mental health services, poor views, and/or relationships with health professionals) were also identified as significant attitudinal barriers to help-seeking (25,49,50).

Age emerged as an important factor associated with help-seeking, but with mixed findings. The study conducted by Calear et al. (32) examined predictors of help-seeking attitudes and intentions and found that older age was associated with more positive views and greater intentions to seek help. In Mackenzie et al.’s (25) study that examined factors associated with past-year mental health service use and perceived need, older age was also found to be positively associated with help-seeking, but only among those who perceived the need for help. In particular, adults aged 75 and older with perceived need were about 8 times more likely than those between ages 55 and 64 to seek help. Yet, in 3 other studies, increased age was negatively associated with service use or help-seeking (49,53,56). In particular, in Blais et al.’s (49) study, older distressed veterans were less likely to utilize mental health services than their younger counterparts, and in the study conducted by Vasiliadis et al. (51), medication use was frequently endorsed among younger rather than older individuals with suicide ideation. In Na et al.’s (58) study, greater age was found to be associated with a less favorable attitude toward suicide which may interfere help-seeking. The effect of age was also identified in research conducted by Dey and Jorm (55) but varied by gender. Specifically, men in the age groups of 45–54 and 55–64 were more likely than those 75 or older to be in treatment, whereas middle-aged females (35–64 years old) were at greater odds of getting treatment compared to those aged 15–24. These findings were supported by other research that found middle-aged adults to be more likely to use any type of mental health services, when compared with younger adults (54). On the contrary, according to medical examiner records, suicide decedents between the ages of 40 and 49 were the least likely age group to disclose suicide intent prior to death (57).

Significant differences in help-seeking by race/ethnicity emerged in 4 studies, with mixed findings. Blais et al. (49) found that non-Hispanic White race (85% of the sample) was associated with decreased utilization of mental health care compared to all other groups. On the other hand, Ko et al. (27) found that non-White racial identity was associated with lower odds of recognizing a need for help-seeking and of receiving behavioral health services from a health professional. Whites in the sample (71.9%) were also more likely to seek help compared to non-White participants. Non-Hispanic Whites were also more likely to use services for suicide ideation (odds ratio [OR] = 2.11), suicide plan (OR = 2.39), and suicide attempt (OR = 2.95) compared to Black, Hispanic, and other groups in Stanley et al.’s study (54). Finally, in Dey and Jorm’s study (55), a higher percentage of non-Swiss participants indicated experiencing suicidality on more than half of the days in the reporting period, and non-Swiss females were at lower odds to be in treatment compared to Swiss females.

Furthermore, several other demographic factors were found to be not associated with help-seeking (ie, neither facilitating nor hindering help-seeking) by multiple studies, including marital status (25,27,49,52,54,58), income (27,49,56–58), and size of the region (27,58).

## Discussion and Implications

This systematic review provides a comprehensive summary of the literature on suicide risk help-seeking behavior among middle- to old-age adults. Findings from this review focused on 3 emergent themes: literature describing the prevalence of service use among various populations (eg, firefighters), prior service use among suicide decedents, and factors influencing help-seeking behavior. The small number of articles included in this review highlights a critical gap in the literature and the need for further study, especially as the older adult population is growing at a rapid rate; Baby Boomers made up over 20% of the U.S. population in 2020, and all Baby Boomers will be 65 or older by 2030 (59). Furthermore, the rate of retirement among this group has accelerated in the era of the coronavirus disease 2019 (COVID-19) (60).

Overall, the prevalence of service use related to suicide risk is relatively low among suicidal adults across this age spectrum. Findings based on national data from Canada and the United States consistently show service use rates are approximately 50% among adults with a history of suicide ideation and/or attempt (35,39,40). This is concerning given that a history of suicide ideation and attempts are significant risk factors associated with subsequent ideation, attempts, and/or death (61). Findings also indicated that service use rates varied by suicide risk history. In general, a greater prevalence of service use was found among individuals with a history of suicide attempts relative to those with suicide ideation but no attempts (40,41). Interestingly, age appeared to play a role in these findings in that older suicide attempters were found to be less engaged in mental health service use (40) than middle-aged adults, particularly among those who were still in the workforce (ie, first responders) (41). Perhaps, for middle-aged adults, supportive work environments, peer and family supports, and/or familial obligations play an important role in influencing help-seeking behaviors. Conversely, older adults may be void of such protective factors. Considering that social connections and feelings of belongingness may begin to diminish in older adults, suicide prevention efforts should consider ways to strengthen support and other protective factors that may help to mitigate suicide risk. Programs that reinforce skills and interests (eg, painting, drawing, and music) can provide meaning and purpose to older adults. Integrating different types of “companion” supports (eg, peer, animal [real and robotic], and online technology) within programs may reduce loneliness and social isolation, key risk factors associated with depression and suicide (62,63). As advances in communication, artificial intelligence, and virtual reality continue to evolve, these supportive forms of technology may enhance social connections and become more relevant and amenable to changing demographics, needs, and preferences of older adults.

On the surface, retrospective studies examining help-seeking behaviors among adults who died by suicide suggest that most (range = 75%–87%) decedents had sought professional help within their last 1–2 years of life. These findings, however, need to be interpreted within the context of “where” or “who” provided care. While a range of professional services was utilized by suicide decedents, such as general practice, psychiatric services, medication, social services, inpatient and outpatient services (43–48), service use visits for mental health problems or suicide risk were low compared to general medical visits, which tended to be higher for middle-aged and older adults due to chronic and physical illnesses associated with age. Similarly, age appeared to play a role in helping-seeking behavior among suicide decedents in that middle-aged adults were more likely to seek professional help for a mental health problem or suicidal thoughts prior to death (43,44), and compared to older adults (46). Various factors may account for older adults’ reluctance to seek help, including feelings of hopelessness, inadequacy, fear of losing one’s independence, and perceived burdensomeness (64,65). Suicide prevention efforts should continue to advocate for the integration of routine screening for suicide risk and/or assessment of suicide risk factors in service settings commonly visited by older adults (eg, primary care, medical clinics, and faith-based organizations). In addition, health care, and community professionals (eg, nurses, home health aides, volunteers, transportation workers, and faith leaders) who have frequent contact with aging individuals should be trained in suicide prevention skills to detect risk and facilitate help-seeking referrals. Examples of training programs include Question, Persuade, and Refer, safeTalk, Counseling on Access to Lethal Means, and Mental Health First Aid.

This systematic review also identified key influencing factors to suicide risk help-seeking. In general, higher suicide prevention literacy, being female, experiencing or having a history of suicidal thoughts and behaviors, and the presence of or higher level of mental health needs were factors associated with greater intention or likelihood to seek help. In contrast, stigma and attitudinal factors, such as having negative beliefs about mental health care or care professionals, emerged as significant barriers to help-seeking (25,32,49,50). Age was also identified as a significant predictor related to seeking help, but with mixed findings; some studies found older age to be predictive of greater service use (25,32), while other studies found the opposite (49,51,53,56). Additionally, 1 study found that middle-aged adults were more likely to seek professional help (54), while another study reported contradicting results (57). More studies are clearly needed to unpack the relationship between age and suicide risk help-seeking. In the context of suicide prevention, particular attention should be given to debunking myths and misconceptions relating to seeking help, providing psychosocial education on the benefits of community supports (especially nonmental health supports that may be more malleable for older adults, men, and high-risk groups such as veterans and first responders), and developing and/or implementing interventions aimed at providing ongoing supportive contact and care monitoring (66) of individuals who experience multiple life stressors and risk factors. Considering that older adults tend to use lethal means with greater planning and intentionality (67), there is a need to develop age-appropriate and culturally responsive prevention strategies to identify suicide risk as early as possible.

### Limitations

There are several limitations within this review. First, as with all systematic reviews, there exists the risk that a relevant study was missed by our selection process. However, we engaged in several practices to enhance the rigor of the review to reduce this risk, including the use of multiple reviewers, triangulation of our search processes (ie, key terms, reference lists, and relevant journals), and a multistage review process guided by PRISMA guidelines (37). Additionally, the search process was iterative and included a second round of searches with adjusted filters, based on initial search findings. Second, this systematic review highlights methodological gaps in the current literature. Twenty-two (*n* = 22) of the 24 articles in this review reported data from quantitative studies, and the majority of these relied on secondary analyses of existing data sets. Furthermore, the studies identified reported data collected in or before 2016. Finally, half of the studies in this review did not provide details on the racial/ethnic make-up of their samples or were unable to conduct analyses along race/ethnicity variables. Future studies are needed to gain a more up-to-date understanding of the prevalence and trends of suicide-related service use among middle- to old-age adults and by specific groups, given the demographic shifts underway and other changes associated with the COVID-19 pandemic. More studies from various countries are also needed to allow for a fine-grained understanding of potential cultural differences in help-seeking across countries. Additionally, future research should engage in community-based participatory research and use qualitative and mixed methods to broaden our understanding of help-seeking attitudes, intentions, and behaviors, as well as influencing factors, among the aging population. Finally, the lack of intervention studies identified in this review reflects an underdeveloped area for future study.

## Conclusion

This systematic review provides a summary of the literature from 2010 to 2020 discussing help-seeking for suicide risk among middle- to old-age adults. The small sample indicates this is an understudied age group within suicide prevention research and highlights a need to explore age-specific experiences of help-seeking for suicide risk. We found rates of service use among middle- to old-age adults to be low in general, and mixed findings related to the role of age on service use for suicide risk specifically. Findings also indicate the history of suicide ideation or attempt, being female, and health literacy as factors associated with higher odds of seeking help. In addition, despite of variations due to specific treatment types and across the life course, racial/ethnic minority groups generally tend to use less services than White adults. Thus, future studies should explore factors shaping low service use among males and racial/ethnic minorities, as well as ways to reduce disparities.

Stigma and negative views about mental health care among older adults appear to inhibit help-seeking. Future studies should build upon this review to develop and test interventions to reduce stigma and increase access and use, especially among aging minorities. This might include antistigma campaigns, educational outreach efforts to widen awareness of risks associated with aging and related life transitions, and expansion of gatekeeper training to prevent suicide. Older adults may be at increased risk as they leave the workforce and enter retirement, and thus prevention and screening efforts should target people during this life transition. This might include, for example, discussions around new sources of meaning, exploration of new roles, and supports to help aging individuals feel connected.

There is also a pressing need to increase suicide prevention competencies across diverse delivery settings, to reach individuals who are less likely to seek help in traditional settings. This includes preparing nonclinical staff to identify risk and encourage referrals for suicide risk screening and assessment. Improving patient–provider communication is also needed to overcome negative perceptions about mental health care services. Given the high rates of suicide ideation and attempts among older adults, it is imperative for future studies to explore the development and implementation of culturally sensitive health care practices aimed to address suicide risk and support suicide risk help-seeking among this aging population.
